# Developing person-centred care in hospices through the voice and leadership of nursing: lessons from the United Kingdom

**DOI:** 10.3389/frhs.2025.1619705

**Published:** 2025-10-15

**Authors:** Erna Haraldsdottir, Marie Cooper, Heather Richardson

**Affiliations:** ^1^ Department of Nursing and Paramedic Science, School of Health Sciences, Queen Margaret University, Edinburgh, United Kingdom; ^2^ The Centre for Awareness and Response to End of Life, St Christopher’s Hospice, London, United Kingdom

**Keywords:** person-centred practice, palliative care, practice development, nurse leadership, hospice

## Abstract

**Introduction:**

Nursing leadership and the voice of nurses are crucial for developing person-centred care in hospices. Concerns have been raised that, as palliative care has evolved from its original vision and become more integrated into the mainstream healthcare system, it has increasingly become medicalised. This paper presents an emancipatory practice development programme aimed at enhancing the visibility and voice of nursing and nurse leadership to improve person-centred care in hospices across the United Kingdom.

**Methods:**

The project was a 10-month collaborative education programme for nursing practice leaders in hospices throughout the United Kingdom, running from September 2023 to July 2024. A total of 24 clinical and practice development leaders from eight different hospices participated. The Kirkpatrick model for evaluating learning programmes was adapted to create a framework for assessing the programme's outcomes and impact. The evaluation process utilised Collaborative Critical Creative Inquiry.

**Results:**

The key findings from the evaluation indicate that the programme created conditions for the participants to gain transformative insights and understanding that positively impacted their practice through emancipatory practice development.

**Discussion:**

The programme enabled leaders of person-centred care in hospices to rekindle their vision for palliative care practice. The participants became more aware of how care was constructed within their organisations and recognised the assumptions that were often taken for granted—assumptions that influenced daily care practices that sometimes leaned towards a traditional medical model. They acquired new skills and knowledge that empowered them to engage more intentionally in making changes to enhance person-centred care.

**Conclusion and implications for practice:**

Humanising healthcare is a global agenda, and within hospice care, nurses are at the heart of transforming care to be more person-centred. They are well-positioned to reclaim the core principles of palliative care, as developed by Cicely Saunders, and push back against the medical model that has overshadowed the development and integration of palliative care into current healthcare systems. Nurses are expert practitioners and leaders who hold positions of authority within their organisations. Yet, for many, their change-making potential is not realised. Innovative learning and development programmes are an essential part of humanising healthcare, and emancipatory practice development programmes can unlock nurses' potential to lead this transformation.

## 1 Introduction

Dame Cicely Saunders, the founder of the hospice movement, humanised care for dying people through her transformative philosophy and principles of care in the 1960s (1). She recognised the limitations of the biomedical model for those who were dying and challenged the overemphasis on disease and the curative focus of the British National Health Service, where death was often seen as a failure. Thus, those who were dying in hospitals were left to die alone as healthcare staff feared death and felt helpless (2). Through the development of the model of *total pain*, Saunders illuminated the needs of dying people and proposed principles of care to meet these needs (3). Her holistic model responded to the suffering of the dying person—not only physical pain but also the emotional, social, and spiritual distress associated with facing one's own death. Saunders' model of care is still regarded as best practice for those with incurable long-term conditions and those who are dying, as recommended by the World Health Organization (4).

Person-centred care is also recognised globally as an essential component of 21st-century healthcare and a vital element in improving the quality of care (5). Scholars within nursing have identified a humanistic orientation as the essence of nursing care, describing it as fundamentally valuing human beings (5).

At its core, person-centred care focuses on the personhood of all those involved in care, with a strong emphasis on healthy and therapeutic relationships—highlighting the relational aspect of care (6). The person-centred framework developed by McCormack et al. was created to operationalise person-centredness in healthcare practice, underpinned by humanistic care theory (7). There is growing evidence that this approach leads to improved health outcomes, particularly for people with long-term conditions (8, 9). The framework has been implemented in healthcare systems worldwide to transform and improve care.

There is a strong alignment between the principles of person-centred care and palliative care nursing, both of which are grounded in the original philosophy and principles of palliative care as developed by Cicely Saunders (1). Both models emphasise a holistic approach that challenges the traditional disease-focused biomedical model of healthcare (10). They share an unwavering focus on what matters to the individual and those close to them. Within this focus lies a moral imperative and a therapeutic intent, expressed through relationships built on effective interpersonal processes (7). This reflects a broader notion of health than the biomedical model—one that embraces all dimensions of human existence and supports living a meaningful life, even in the face of death (11).

While it is widely acknowledged that these two models of care are often better suited to meeting the needs of people requiring palliative care, it remains challenging to fully embed them in practice (10). One reason for this is the ongoing medicalisation of healthcare and the entrenched hierarchy of the traditional biomedical model (12).

Nurses are uniquely positioned to challenge this hierarchy and lead the implementation of care that is driven by person-centred palliative principles. The theoretical and practical orientations of nursing align closely with the humanist perspective of Cicely Saunders and the person-centred care theory developed by McCormack et al.

Nursing leadership and the voice of nurses are key to the development of person-centred palliative care in hospices (13). Nurses represent the largest regulated healthcare professional workforce delivering palliative care across a range of clinical settings (14). They have diverse roles and responsibilities, and the caring and artistic dimensions of nursing are fundamental to palliative and end-of-life care (15). However, concerns have been raised that, as palliative care has evolved and become integrated into mainstream healthcare, it has become increasingly medicalised due to the inherited hierarchy of the biomedical model (16). This has led to a lack of visibility of the nursing contribution to palliative care and diminished recognition of their essential role. There is concern that the medicalisation of palliative care has silenced the voice of nursing and diluted the artistic elements of nursing practice (14, 15), resulting in a less articulated contribution to care (15).

The purpose of this paper is to present findings from an Emancipatory Practice Development (ePD) programme. The programme focused on developing and strengthening the visibility and voice of nursing and nurse leadership to enhance person-centred care in hospices across the United Kingdom.

### 1.1 Context

The practice development project was a 10-month collaborative education programme for leaders of nursing practice in hospices across the United Kingdom (see Table 1). Participants included 24 clinical and practice development leaders from eight hospices—two in Scotland and six in England—who held pivotal roles in leading and implementing direct care and/or driving change and improvement within their organisations (see Table 2). Hospices were selected to ensure geographical diversity and were invited to participate in the project.

**Table 1:** Overview of the themes of each day during the 7 days of the course.

Day 1	• Appreciate the value of Practice Development as an approach • Recognise its contribution to outcomes—for patients, nurses, the wider multidisciplinary team (MDT), and the organisation • Understand the place of practice development in enabling person-centred practice/nursing care • Create an intention to translate the theory of practice development (PD) into practice • Offer tools/approaches to support the development of the practice of nursing
Day 2	• Appreciate what a flourishing culture looks and feels like—for patients, professionals, and the organisation • Recognise the relationship between a flourishing culture and the nurse who is flourishing in their role • Appreciate how organisational culture will shape the culture of care • Recognise structures and processes that contribute to a flourishing culture • Identify behaviours that support and confirm a flourishing culture
Day 3	• Appreciate the different elements of/contributors to person-centred practice • Recognise the contribution of nursing to person-centred practice and what is unique to nursing within the wider MDT • Appreciate the place of values and beliefs held by individuals and organisations in shaping practice • Acknowledge how the culture of the unit/organisation supports or inhibits person-centred practice • Recognise the value of a shared vision as a starting point to the journey of practice development • Become familiar with the journey of practice development as a basis for an organisational action plan
Day 4	• Appreciating what contemporary transformational leadership looks like • Feeling confident to utilise a coaching approach in the development of others • Appreciating the talents across a team to achieve person-centred care
Day 5	• Appreciate how/where nurses can most effectively contribute to positive outcomes for patients, each other. and the organisation through their practice • Recognise the value of stories of nurses' practice to help identify key moments in care and how they are best enhanced • Identify what needs to be protected, enhanced. or introduced in terms of nursing practice to improve or maintain the quality of care • Consider how the profession of nursing is advanced at the local level to ensure its appropriate impact
Day 6	• Test the value of a theory of change in shaping intention, plans, and evaluation • Explore ways of maintaining ambition and momentum beyond initial enthusiasm • Recognise the place of personal and professional leverage to support change • Identify how a practice development approach fits into broader strategies for improvement in care and its evaluation • Create a story that requests long-term local investment in this work
Day 7	• Reflect on the progress and challenges of individual hospices as a basis for driving person-centred palliative nursing—through further learning and financial and other investment/support in this work at the national and organisational levels • Review the details of the programme and the experience of learning as a basis for developing it further • Describe future support required from each other, CARE, academic leaders, and beyond • Confirm offers of talent and support to the community • Establish community of practice (as required)

**Table 2:** Number and role of participants from each hospice.

Hospice no.	Participants’ roles
1	Staff Nurse; Head of Wellbeing Service; Ward Team Leader
2	Ward Sister; Clinical Service Manager; Senior Staff Nurse
3	Consultant Nurse; Ward Manager; Deputy Ward Manager
4	Practice Development Facilitator; Quality Lead; Charge Nurse; Staff Nurse
5	Charge Nurse; Nurse Manager; Inpatient Unit
6	Matron; Nurse, Inpatient Unit; Clinical Lead; Inpatient Unit
7	Assistant Director of Service; Ward Sister; Clinical Lead; Inpatient Unit
8	Clinical Service Manager; Ward Sister; Senior Staff Nurse

Invitation emails were sent to the nursing care leaders within each hospice, offering the opportunity for two to three members of staff to take part. The eligibility criteria required participants to hold a role in practice development or serve as an operational manager of nursing care. One hospice team withdrew from the programme midway due to time constraints and organisational changes, although they actively participated during the first half.

The programme comprised 7 workshop days delivered over a 7-month period. Of these, two workshops were held face-to-face—one at the beginning and one at the end of the project.

Each hospice received mentorship from one of three facilitators throughout the programme. All materials were hosted on a password-protected online learning platform, accessible only by participants and facilitators. A WhatsApp group was also created for those who wished to maintain direct contact during the programme.

The programme was designed to be flexible, allowing each participant to develop a contextualised practice development plan tailored to their own hospice setting.

### 1.2 Underpinning theory of the programme

ePD was the foundational approach to learning and development within the programme. Over the past two decades, ePD has evolved as a continuous process for cultivating person-centred cultures and care (17). Accordingly, the programme was designed with a flexible and reflective structure, enabling participants to gain insights and knowledge relevant to their personal and professional development and their specific contexts.

The ultimate purpose of the programme was to create conditions that would enable participants to develop the knowledge and skills necessary to transform care to be more person-centred. Through the lens of ePD, the focus was placed on the culture and context of care within each participant's practice area, with the responsibility for action falling to the participants themselves (18). The aim was to support the development of the participants' personhood, values, and beliefs as the foundation for their practice development and learning (19), ultimately enabling them to become person-centred facilitators in their own settings.

The programme remained true to the creative and reflective nature of ePD, recognising these as critical elements for transformation. The approach is grounded in critical social science, which is underpinned by the principles of enlightenment, empowerment, and emancipation (20). This involves raising consciousness, motivating participants to take action, and ultimately engaging in transformative practice (21).

Critical theory has been increasingly applied to healthcare research over the past two decades (22). As a philosophical approach, it challenges taken-for-granted assumptions, questions self-evident realities, and critiques unexamined policies, practices, and procedures. It explores power relations, knowledge formation, and claims to truth, offering tools to critically analyse ideological positions—an approach highly relevant to the findings of this evaluation.

Emancipatory Practice Development also draws on Habermas' dualist theory of society, which distinguishes between the lifeworld (the realm of human experience and meaning) and the system (the realm of economic and technological structures) (22). This theoretical positioning warns against reducing human experience to technical or economic considerations. In this programme, we intentionally focused on the lifeworld—i.e., the lived experiences of participants—rather than on technical skill acquisition alone.

The ePD approach fosters learning that is grounded in the realities of practice. It differs from traditional technical approaches by prioritising critical reflection and the development of the individual as a change agent. The aim is to support participants in identifying the gap between current and desired practice and to explore what is needed to bridge that gap to enable more person-centred care (23). The core focus of the programme was to support the development of new insights and understandings that are transformative in nature (17).

Facilitation is a core principle of ePD. It supports individuals and teams in understanding the context in which they work and identifying the characteristics of that context that may contribute to the gap between the current and desired practice. Thus, the central site of learning is everyday practice, with the goal of enabling meaningful change (17).

To further support participants, the Lantern Model of palliative care nursing was introduced as a theoretical framework. Developed by two of the authors (HR and MC), the Lantern Model highlights the specific skills and knowledge required in palliative care nursing (24, 25). It builds on the Person-Centred Practice Framework developed by McCormack et al. (7) and is rooted in the philosophy and principles of Dame Cicely Saunders. The Lantern Model adapts the person-centred framework to focus more specifically on the context, knowledge, and skills relevant to palliative care nursing in contemporary health and social care. It also reclaims Saunders' original vision as a guiding theoretical foundation for palliative care (24, 25).

## 2 Evaluation framework and methodology

We adapted the four-stage Kirkpatrick Model (26) to evaluate the outcomes and impact of the teaching programme, focusing on reaction, learning, and behaviour. This model is widely used to assess educational programmes (27). In our case, it provided a framework for presenting findings across four adapted levels: perceptions and attitudes, new insights and understanding, impact on behaviour in the workplace, and impact at the organisational (macro) level.

The analytical evaluation process was informed by Collaborative Critical Creative Inquiry, as developed by McCormack and Titchen. This approach allows for the integration of diverse datasets, including those derived from arts-based methods (28). Data collection was conducted over the course of the programme and aimed to gain an in-depth understanding of participants' experiences and the meaning they attributed to them.

### 2.1 Aims

The overall aim of the evaluation was to assess whether the programme had a transformative impact on the participants, as intended. The central research question was as follows:

How does a person-centred palliative care development programme enable nursing leaders within a hospice context to grow and develop practice?

### 2.2 Ethical considerations

The evaluation was underpinned by a robust ethical framework aligned with the principles of person-centredness and embedded throughout the programme's delivery and evaluation. The key ethical considerations included the following:

• Ensuring voluntary participation,
• Maintaining anonymity and confidentiality, and
• Promoting psychological safety throughout the process.

The participants were informed that the evaluation would run in parallel with the programme and be embedded within it. Ways of working were established at the outset, with consistent “check-ins” throughout. For these reasons, formal ethical approval was not sought, as the evaluation was considered an integral part of the educational programme.

### 2.3 Data collection and analysis

The Collaborative Critical Creative Inquiry method allowed for multiple forms of data to be collected throughout the programme (28), focusing on the participants' reflections and descriptions of “in-the-moment” experiences. These reflections captured the participants' perceptions as they engaged with the development process.

To support this, reflective aids were used during workshops, including a Claims, Concerns, and Issues session, where the participants critically reflected on their own practices and the influence of their organisational context (29). This enabled them to identify areas of strength, concerns, and priorities for change. The format was based on Fourth Generation Evaluation (29).

Narrative writing was used to surface stories from practice, highlighting both positive examples and areas requiring improvement (30). Participants were also introduced to Haiku writing as a creative method for expressing their feelings and progress. While the traditional five-seven-five syllable structure proved restrictive, this was adapted into more flexible short poems of three to four lines (31).

An evaluation workshop was held on the final day of the programme, focusing on the enablers, challenges, and achievements throughout the change process. Each activity within the programme served a dual purpose: supporting individual and group learning and simultaneously generating data that reflected significant insights, learning, transformation, and outcomes.

Analysis was an iterative process embedded within the programme. It was guided by hermeneutic and interpretive approaches (18) and was conducted collaboratively with all the participants during the final workshop. This included a reflective exercise based on the following questions: “What do I see?”, “What do I feel?”, and “What do I imagine?”

## 3 Results

Four key themes emerged from the analysis and were aligned with the adapted Kirkpatrick evaluation framework. They are as follows:

• Perceptions and attitude change—exploring how the programme supported personal growth and shifts in mindset.
• New insights and understanding—identifying new competencies and knowledge gained through participation.
• Behavioural change in the workplace—examining how participants translated learning into practice.
• Impact at the organisational (macro) level—assessing broader systemic influence within the hospice setting.

In presenting the findings and the subsequent discussion, we will refer to the development of leaders in person-centred palliative care nursing practice within hospice organisations.

### 3.1 Perceptions and attitude change

The participants reported a strong sense of rekindling their vision for palliative care nursing practice during the programme. This renewed clarity and purpose translated into a deeper commitment to person-centred care and a strengthened engagement with practice development in their own settings. It was also evident that the programme enabled the participants to develop a clearer sense of themselves as leaders in person-centred palliative care.

#### 3.1.1 Re-engaging with values and beliefs

**Figure 1:**
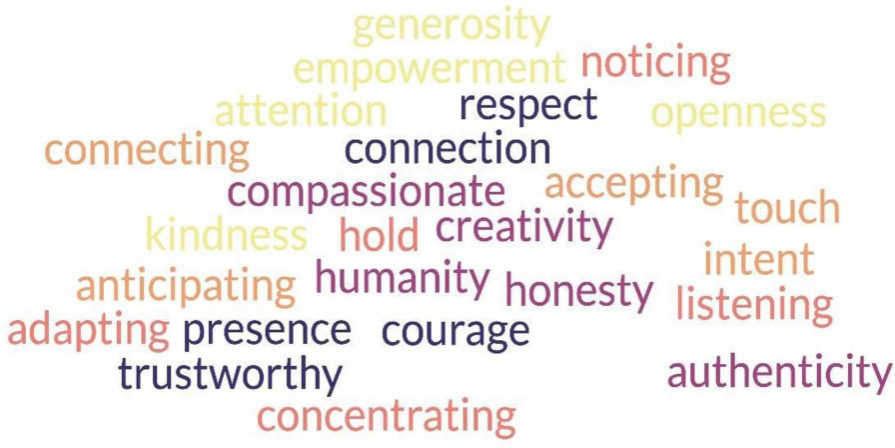
One word description from particpants capturing the essence of palliative care nursing.

The participants valued the opportunity to reflect on and share their personal values and beliefs in relation to nursing practice, particularly in connection with the original philosophy of palliative care as developed by Dame Cicely Saunders. This reflective process helped surface a shared vision of desired practice and highlighted the creative and relational nature of palliative care nursing. It also deepened the participants' understanding of the role of interpersonal skills as a core element of nursing expertise and contribution. Figure 1 demonstrates a word cloud displaying key themes related to caregiving qualities and values as seen by participants.

The programme's structure, which brought together participants from different hospices, fostered a strong sense of shared values and vision. This created a community of practice that supported mutual learning and a collective sense of purpose. The following poems, written by participants, reflect this shared experience:

**Poem 1 (p7)**

Inspiring change together

Learning, growth, development

Exciting future

**Poem 2 (p3)**

Working together,

meeting new people,

united by a common aim

#### 3.1.2 Rekindling the commitment to person-centred nursing practice

The shared vision and purpose cultivated during the programme created the conditions for the participants to reconnect with the unique contribution of palliative care. This was further supported by a focus on the positive impact of person-centred practice within the hospice context. Participants shared stories of good practice, which helped make their contributions more visible, better articulated, and appreciated, as evidenced by the following quote:

“I loved how we started the day with an exercise on positive reflection- so often we are asked to focus on “what went wrong” or reviewing the negative.” (p7).

Narratives shared from practice during the programme enabled participants to deepen their insights and gain greater clarity around the unique contribution of person-centred practice within the hospice setting. This was particularly evident in the short poems created by the participants, which reflected their evolving understanding. In addition, a new and critical awareness emerged around the use of language, namely, its role in articulating practice and fostering connection. This recognition brought a renewed sense of energy and purpose to the participants' leadership and practice development.

**Poem 3 (p6)**

Stories are our strength

The best way to share

Impact

Power of Sharing

**Poem 4 (p2)**

Language is vital

Open our minds to hope

Share, Care, Grow, Flourish

It was evident that the programme enabled the participants to develop deeper insights and a heightened awareness of the unique contribution of person-centred palliative care within hospice settings. The narrative data demonstrated how viewing practice through the lens of the Lantern Model provided the participants with a language to articulate aspects of their work that are often hidden or poorly expressed. This gave them a new voice to make their contributions visible—contributions that are frequently taken for granted in the routines of daily practice. As a result, the participants were able to celebrate and acknowledge their role, which brought renewed energy and inspiration to their leadership in practice development.

### 3.2 Development of insights and understanding

The analysis demonstrated that the programme enabled the participants to gain new insights into and an understanding of their roles and of practice development. They became more able to recognise incremental change, even when progress was slow or subtle. It was also evident that the participants developed a heightened awareness of their role within the organisation, and a deeper appreciation of its essence and significance. Furthermore, there was a newfound confidence and resilience in viewing practice development as an ongoing journey—one marked by both high and low points, but sustained by purpose and reflection.

**Poem 5 (p5)**

Transformable train, running down the tracks

Many miles to go

Seeds of change will grow

**Poem 6 (1 1)**

Empathizer, Catalyst; Storyteller. Coach.

So many strengths to harness

To Shape

To Lead

**Poem 7 (1 9)**

Proud of where we are

Small steps…

A transformation.

A lightbulb moment

It was evident throughout the programme that the participants felt supported in their role as practice developers, which in turn enabled their personal and professional growth. This is reflected in the following quote from one participant:

“Feeling supported in the role of practice development, I feel clearer about my strengths—energised, excited, and aware of the many possibilities ahead. New understanding and insight into the role of practice development are demonstrating transformation.” (p21).

Alongside this sense of empowerment, participants also expressed an appreciation of the challenges inherent in the role. One participant noted the following:

“Understanding [the need for change] will take time. [There is a need for] being visible enough in the midst of lots of ‘other things’.” (p17).

### 3.3 Impact on behaviour in the workplace

The analysis highlighted that the participants began engaging with greater intentionality in making changes to enhance person-centredness in their practice following the programme. This was evident in how they applied newly acquired knowledge and approaches within their workplace settings, as demonstrated by the following quote:

“I am much more creative in my practice now—and even using poetry!” (p15).

Many of the participants adopted methods introduced during the programme, such as storytelling and positive reflection, to illuminate good practice and foster a culture of appreciation and learning. One participant stated the following:

“Starting with a ‘good care’ reflection—this is something that we are using now with our registered nurses.” (p11).

The participants also recognised the importance of developing a shared vision and clearly articulating the values and beliefs underpinning person-centred care. Several facilitated workshops within their own teams to promote this focus, with one participant stating the following:

“I will continue wider team engagement to clarify aims and our shared vision.” (p8).

The programme fostered a stronger sense of role clarity and commitment to practice development within hospices. The participants became more intentional in their actions to influence and shift organisational culture, as evidenced by the following quote:

“I need buy-in from our team in understanding how the micro actions affect the macro environment.” (p21).

### 3.4 Impact on the macro level within the participants’ organisations

The analysis revealed potential macro-level impact stemming from individual transformation during the programme. Most of the participants felt that being part of a community of learners had strengthened their vision and resilience. They reported feeling better equipped to face challenges and setbacks in their roles, including efforts needed “to raise awareness of person-centred development within the hospice” (p7) and to “open their organisation's eyes to new opportunities” (p11).

Despite this, participants expressed concerns about the implementation of change within their practice environments and questioned how well their organisational contexts were prepared to support transformation, as evidenced by the following quotes:

“How do I carry these conversations [from the programme] into the hospice?” (p10).

“How do I get engagement from the nursing team and help others come on the journey of change?” (p2).

“How do I get buy-in from the team and senior managers, and keep the momentum and engagement in practice?” (p6).

On the final day of the programme, the participants worked in hospice-based teams to plan future intentions. They identified small-scale initiatives that could support the transformation towards more person-centred care. Many of these were micro actions with the potential to influence the macro system. Examples included the following:

• Facilitating workshops within their hospices based on learning from the programme,
• Revisiting the structure and format of multidisciplinary team meetings to enhance language and focus on person-centredness. and
• Promoting and implementing the Lantern Model as a framework for enhancing person-centred care, with some participants already initiating education and awareness activities around the model.

## 4 Discussion

Emancipation refers to the process of setting individuals free from unexamined, taken-for-granted assumptions and expectations (21). Throughout the programme, the participants were guided to critically reflect on and bring to light such assumptions—both personal and organisational—that may hinder the development of person-centred practice. The programme created opportunities to question established norms and routines within hospice care, many of which had previously gone unchallenged.

McCormack et al. describe programmes of this nature as creating a brave space—a psychologically safe yet challenging environment that enables effective transformation of practice (36). The findings from our project demonstrate that the programme successfully created such a brave space, where participants experienced meaningful transformation.

The following discussion explores this transformation through the lens of emancipation, focusing on the following three key themes that represent the mechanisms of change:

• Professional ideology brought to light,
• Practice knowledge brought to light, and
• Emancipatory practice brought to light.

### 4.1 Professional ideology brought to light

The participants were able to identify elements of behaviour and actions within their practice that reflected a person-centred orientation—values they strongly believed in but had not previously recognised or articulated. These aspects of their professional identity had remained hidden prior to the programme and were brought to light through reflective learning. This realisation fostered a sense of confidence and excitement about their practice, the care they provided, and its impact.

Through the programme, the participants gained a new understanding of their professional ideology. Ideology refers to the shared meanings and values of a group; it is socially constructed and does not exist as an objective truth but is made visible through the behaviours and actions of its members (32). It became evident that the ideology of the palliative care nurses and leaders—particularly those leading person-centred practice development—aligned closely with Cicely Saunders' philosophy, as articulated in the Lantern Model of Palliative Nursing (24, 25). Awareness of this alignment strengthened the participants' professional identity, which had previously been unspoken and obscured.

Trede et al. (32) argue that when professional ideology remains hidden, it is often because another profession has gained authority, social status, and dominance. Within traditional healthcare systems, the medical-biological model holds an authoritative position, shaping practice and discourse. James and Field (33) highlight how the hospice movement became over-medicalised as it was integrated into mainstream healthcare. Power and domination within organisations influence who determines actions, what topics are discussed or avoided, and who has a voice in decision-making (34).

By raising awareness of power dynamics and ideological bias, the participants became more attuned to how authority was exercised within their organisations and how this influenced practice. They recognised that unexamined ideological biases could hinder the development of person-centred care. Identifying and challenging these biases became a vital part of their role as leaders in care transformation.

More than two decades ago, McCormack et al. (17) emphasised the importance of attending to cultural and contextual factors in humanising healthcare. Without this focus, they argued, the system cannot truly place the person at the centre of care. McCormack also called for a revolution in healthcare education, advocating for programmes that prioritise human elements alongside technical development (12).

More recently, Cook et al. called for innovative approaches to healthcare education, promoting congruence between education and practice to support the transformation of care and the humanisation of healthcare (35). Despite these calls, educational programmes have been slow to shift from a technical focus to more transformative and emancipatory approaches.

There is a need for organisational bravery among nurse practitioners and leaders to invest in learning and teaching programmes that enable teams to step back from routine practice and reflect on the deeper meanings attached to their work (36).

### 4.2 Practice knowledge brought to light

During the programme, the participants came to recognise that their approach to care was deeply rooted in moral and ethical intent. This realisation was significant, as they began to see how their values aligned strongly with person-centred practice and Cicely Saunders' philosophy of palliative care. These insights helped the participants understand that both models of care emphasise this orientation, which Saunders formulated as the expert practice knowledge essential for palliative and end-of-life care (1).

Throughout the programme, the participants also realised that the knowledge they considered central to their expertise was often subjective, embedded in experience, and taken for granted. The programme created conditions for consciousness-raising, enabling the participants to become aware of how their actions and behaviours were guided by deep, experiential knowledge. They began to recognise the knowledge base they operated from and its contribution to positive health outcomes for those they cared for.

This form of knowledge aligns with what Habermas described as practical knowledge (37). Habermas proposed that all knowledge is shaped by personal and professional interests and can be categorised into three distinct domains: technical, practical, and emancipatory. Each domain generates different types of knowledge, poses different questions, and influences different actions and perceptions of reality.

Practical knowledge is “stored” knowledge that guides practitioners' actions. It involves understanding intentions, meanings, values, and interests—both one's own and those of others—and is based on a reflexive and evolving understanding of the situation in which one is practising. Within nursing, this has been described as practice wisdom and professional artistry (38).

In contrast, technical knowledge, as defined by Habermas, is driven by a desire for control, prediction, and certainty. This aligns with the traditional biomedical model of healthcare, which prioritises efficiency, measurable outcomes, and curative approaches.

While Habermas argued that both types of knowledge are equally important (37), the modern healthcare system has inherited a hierarchy of knowledge that places technical knowledge—rooted in biomedical science—above practical and emancipatory knowledge. In the context of humanising healthcare, it is essential to challenge this hierarchy and recognise the value of practical knowledge and wisdom as equally vital.

Indeed, it is this form of knowledge that enables care to meet the needs of many individuals, particularly those with complex, long-term, or end-of-life conditions—as Cicely Saunders identified in the 1960s. The traditional biomedical model is increasingly ill-suited to address the challenges posed by an ageing population and the growing burden of chronic illness.

Person-centred practice development seeks to humanise healthcare by valuing both technical expertise and practice wisdom, ensuring that care is not only clinically effective but also ethically grounded, relational, and responsive to individual needs.

### 4.3 Emancipatory practice brought to light

The programme created conditions for raising awareness of the nature and importance of practice-oriented knowledge. This enabled the participants to recognise the need for applying the third type of knowledge described by Habermas—emancipatory knowledge. For Habermas, this form of knowledge is driven by the desire for liberation from unnecessary constraints and limitations (37). Emancipatory practice emerges when care is guided by less hierarchical, critically examined knowledge, and when the values and voices of all involved, including the patient, are treated as equal.

This orientation is foundational to Cicely Saunders' development of palliative care in the late 1950s and early 1960s, which challenged the dominance of technical knowledge in healthcare. Saunders responded by advocating for a model of care grounded in practice wisdom, relational expertise, and ethical intent (2).

Over recent decades, concerns were raised about the over-medicalisation of palliative care, particularly as it transitioned from its grassroots origins into mainstream healthcare systems (33). The programme enabled the participants to reflect on their own values and intentions, and to recognise when these were misaligned with the organisational values driving day-to-day practice. This critical awareness led to new insights into how organisational culture could either hinder or facilitate person-centred care, challenging the medicalisation of palliative care.

Throughout the programme, the participants gained a deeper understanding of how their roles involved challenging existing cultures and aligning with an emancipatory orientation to knowledge. They recognised their responsibility in addressing the imbalance within the hierarchy of knowledge, where technical expertise often overshadows relational and ethical dimensions of care. Emancipatory practice in palliative care embraces the complexity of practice, where facts, technical knowledge, values, and professional wisdom co-exist and inform one another.

The participants also developed greater insight into the need for cultural change, recognising their role in navigating and leading this journey. They became more aware of their organisational structures and contexts, and how these influenced the potential for transformation. Importantly, they acknowledged the emotional and professional demands of their role, including the need for self-care and resilience.

The programme helped the participants reframe change and transformation as a process or journey, rather than a series of immediate outcomes. This perspective enabled them to appreciate incremental progress and to develop new ways of measuring success through small but meaningful steps. See summary of key findings in Table 3.

**Table 3:** Summary of the key findings.

Theme	Philosophy	Model of care	Key insights	Implications for practice
Perceptions and attitude change	Critical theory	Cicely Saunders’ model of palliative care	The participants rekindled their vision for person-centred palliative care and developed a stronger sense of identity as leaders	Strengthens leadership confidence and commitment to person-centred care
	Habermas’ emancipatory practice development			
		The Lantern model		
Practice knowledge brought to light	Critical theory	Cicely Saunders’ model of palliative care	The participants recognised the value of moral, ethical, and experiential knowledge, often hidden in routine practice	Validates practice wisdom and highlights the need to challenge the dominance of technical knowledge
	Habermas emancipatory practice development			
		The Lantern model		
Emancipatory practice brought to light	Critical theory	Cicely Saunders’ model of palliative care	The participants became aware of organisational assumptions and power dynamics that hinder person-centred care	Encourages cultural change and critical reflection within hospice organisations
	Habermas’ emancipatory practice development			
		The Lantern model		
Behavioural change in the workplace	Critical theory	Cicely Saunders’ model of palliative care	The participants applied new methods (e.g., storytelling, poetry, and reflective practice) and facilitated team engagement	Demonstrates practical application of learning and potential for culture shift
	Habermas’ emancipatory practice development			
		The Lantern model		
Macro-level impact	Critical theory	Cicely Saunders’ model of palliative care	The participants initiated small-scale actions with the potential to influence organisational culture	Highlights the need for follow-up to assess long-term organisational transformation
	Habermas’ emancipatory practice development			
		The Lantern model		

## 5 Conclusion

This evaluation has demonstrated how an emancipatory practice development programme supported meaningful transformation among its participants. A key outcome was the development of increased confidence and strengthened intention in their roles as leaders of person-centred practice development within hospices. This transformation was underpinned by a growing awareness of their professional ideology, which had previously been suppressed by the dominant biomedical hierarchy embedded in healthcare systems.

The mechanism for this transformation was twofold. First, the programme enabled the participants to rekindle their vision for person-centred palliative care and to recognise the value of their practice knowledge, which is often overshadowed by the medical model. The participants gained clarity about their unique contributions and the impact of person-centred care on those they support. They also realised that the skills and knowledge they relied on were often embedded, taken for granted, and not visibly acknowledged. The Lantern Model provided a tangible framework for articulating and celebrating this expertise.

Second, the programme helped the participants become more aware of how care was constructed within their organisations and how taken-for-granted assumptions shaped daily practices. These assumptions, often biased and unexamined, were identified as barriers to person-centred care and became focal points for challenge and change.

This paper has outlined the underpinning mechanisms that support transformation within an emancipatory practice development programme. However, a limitation of this project is the lack of follow-up to assess the organisational-level impact of the programme. While the findings clearly demonstrate the programme's potential to transform individual leaders and practitioners, we were unable to track specific change initiatives or measure their outcomes within hospice settings.

Future evaluations should consider incorporating longitudinal follow-up to explore how individual transformation translates into organisational change and the sustained development of person-centred care.

## Data Availability

The datasets presented in this article are not readily available because the data are confidential. Requests to access the datasets should be directed to eharaldsdottir@qmu.ac.uk.
